# Expectations and motives for nominating a family caregiver to partake in a web-based psychoeducational intervention: perspectives of patients with advanced illness in palliative care

**DOI:** 10.1007/s00520-026-11105-y

**Published:** 2026-08-20

**Authors:** Sandra Doveson, Viktoria Wallin, Ulrika Kreicbergs, Anette Alvariza

**Affiliations:** 1 The Department of Health Care Science, Marie Cederschiöld University, Box 11189, 100 61 Stockholm, Sweden; 2 Department of Nursing Science, Sophiahemmet University, Stockholm, Sweden; 3 Louis Dundas Centre, Great Ormond Street Institute of Child Health, University College London, London, United Kingdom; 4 Department of Women’s and Children’s Health, Karolinska Institutet, Stockholm, Sweden; 5 Research and Development-Unit/Palliative Care, Stockholms Sjukhem, Stockholm, Sweden

**Keywords:** Cancer, Family caregiver, Home care services, Internet-based intervention, Oncology, Palliative care

## Abstract

**Purpose:**

The field of support interventions targeting family caregivers in palliative care is growing, yet little is known about the patient’s perspective when a family caregiver partakes. This study explored what patients in specialized palliative home care expected from, and what motives they had for nominating a family caregiver to partake in, a family caregiver-targeted web-based psychoeducational intervention.

**Methods:**

This qualitative study is part of a randomized controlled trial evaluating the effects of the family caregiver-targeted web-based psychoeducational intervention “narstaende.se.” Patients were recruited from specialized palliative home care services after their family caregiver had participated in the intervention arm of the trial. The present study had a qualitative approach using an interpretive descriptive design. Twenty-two interviews were conducted.

**Results:**

The patients nominated family caregivers out of concern and recognition of the burden they faced and wished for the family caregivers to be offered support. The patients’ choice was mindful, and they refrained from nominating persons whom they perceived as too frail or busy. They expected the intervention would encourage conversations about emotions, illness and the future.

**Conclusion:**

Patients with life-threatening illnesses enrolled in specialized palliative home care nominated their family caregiver for a family caregiver-targeted web-based psychoeducational intervention out of love, guilt, and responsibility. They expected to approach topics that had previously been avoided and could see potential benefits also for themselves. The study reinforces the implications for support for family caregivers when organizing and providing care for patients with life-threatening illness.

**Trial registration:**

NCT05785494 at clinicaltrials.gov (registered on 2023.02.07).

## Background

Patients with advanced cancer and other life-threatening illnesses receiving palliative care in their own home as opposed to the hospital, even at the end of life, has become increasingly common and the trend in Sweden moves towards more home deaths [1]. Being at home at the end of life is also preferred by many [2, 3]. However, the preference to be cared for in one’s own home is also associated with cohabitation [3], which underlines the important role of family caregivers in palliative care. Not only are family caregivers often involved in the patient’s care, but their physical and psychological morbidity are also interdependent with that of the patient [4]. This shows how the relationship between patient and family caregiver is mutual and one’s respective experiences affect the other’s. A large body of research has shown the struggles of, and impact on, family caregivers of patients with life-threatening illness [5–7], urging for interventions to support them in their situation. The landscape of research on interventions aimed at supporting family caregivers is constantly expanding and today there are a large number of interventions described [8, 9]. The growing number of web-based interventions [10–12] have also made supportive interventions increasingly available for family caregivers who might struggle to attend in-person meetings.

It has been shown that patients in palliative care partake in research with the hopes of contributing and helping others [13]. Similarly, family caregivers of patients in palliative care choose to partake in research hoping to improve care and share their experience [14]. In research, access to family caregivers is most commonly obtained via the patient, who chooses one or several family caregivers for the researchers to contact about study participation. Though implications for participating in research have been described by both patients and family caregivers, patients in palliative care can still hesitate or choose not to nominate their family caregiver for participation to protect them from the burden [15]. There is, however, a lack of research that focuses on the patient perspectives when family caregivers are offered participation in research studies. Hence, the aim of this study was to explore what patients in specialized palliative home care expected from, and what motives they had for nominating a family caregiver to partake in, a family caregiver-targeted web-based psychoeducational intervention.

## Methods

### Design

The study had a qualitative approach using an interpretive descriptive design [16].

### Study context—the overarching randomized controlled trial

**Fig. 1:**
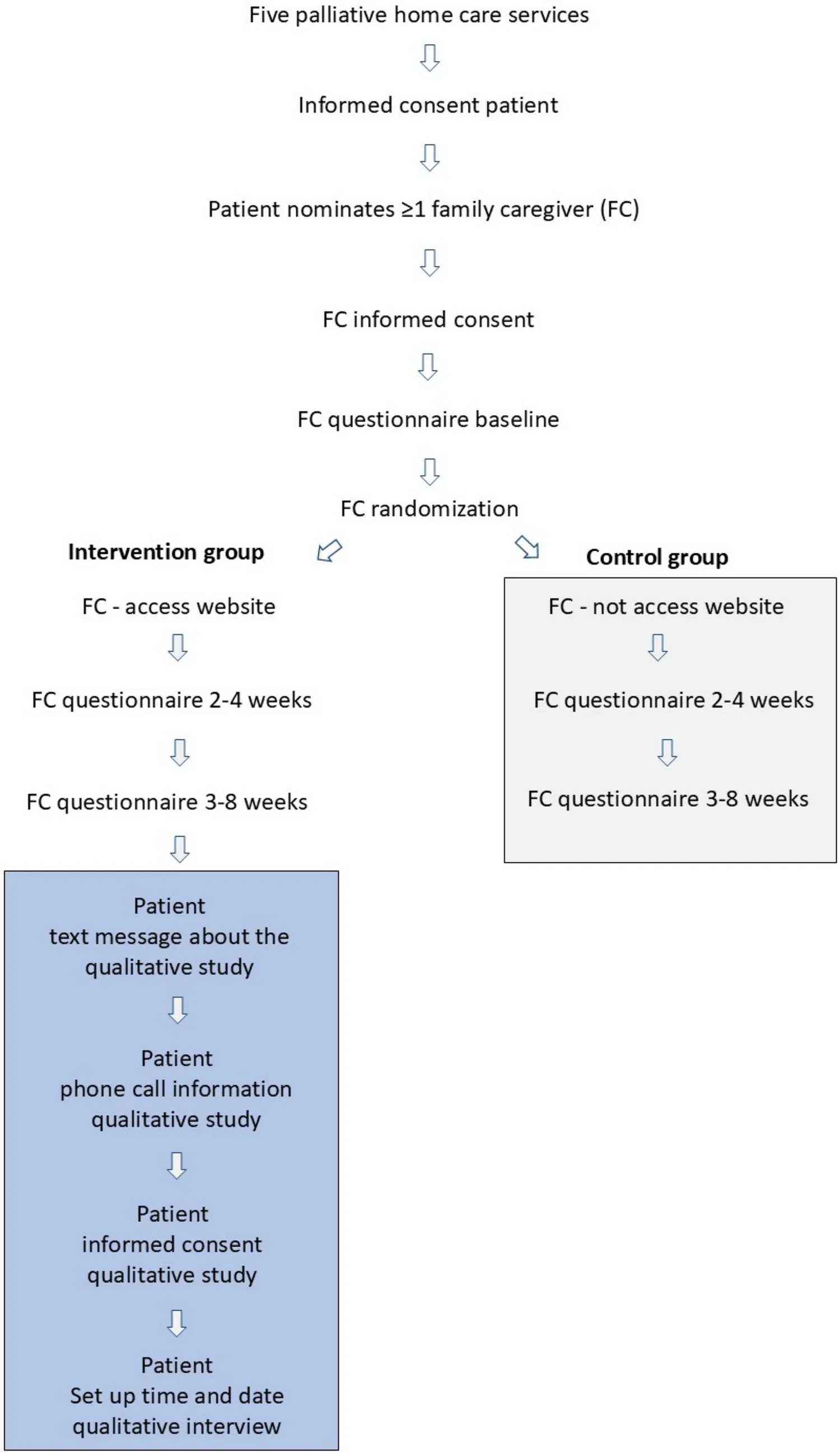
Overview of the study inclusion process

The present study is part of a randomized controlled trial (NCT05785494 at clinicaltrials.gov) evaluating the effects of the family caregiver-targeted web-based psychoeducational intervention “narstaende.se” (“narstaende” translates approximately to “family caregiver” in English). The intervention targets preparedness for caregiving and preparedness for death among 205 (103 intervention and 102 control) family caregivers in specialized palliative home care services [12]. The intervention is presented at a website and comprises 23 videos, supplementary texts, links to useful resources, and a moderated chat forum [17]. The videos and texts are organized into three domains: 1: *support for you—being a family caregiver*; 2: *how to give support*; and 3: *talk about it*. Family caregivers of patients with life-threatening illness and palliative care needs were recruited from five specialized palliative home care services in an urban part of Sweden (Fig. 1). The palliative home care services offer individually tailored round-the-clock care for adult patients residing in their own homes while living with complex illnesses—mainly advanced cancer but also congestive heart failure, chronic obstructive pulmonary disease, or neurological illnesses.

### Procedure and participants—this qualitative study

Patients for the present qualitative study were recruited after their family caregiver had participated in the intervention arm of the randomized controlled trial and hence, had access to the web-based intervention (Fig. 1). Thirty patients were invited consecutively, meaning they were contacted in the order their family caregiver had completed their time in the study. When the present qualitative study commenced, the first patient asked was the one whose family caregiver had most recently completed their final study questionnaire and then we proceeded with the second-to-last patient and so on. The patients were sent a short text message explaining that the study was ongoing and that a researcher would call them in the next few days. They were then telephoned by the first author who informed about the study and asked whether they were interested in participating. Twenty-two patients agreed to participate. Eight patients declined due to not feeling well enough, not having the time for it or did not answer the phone despite two calls on different days.

### Data generation

**Fig. 2:**
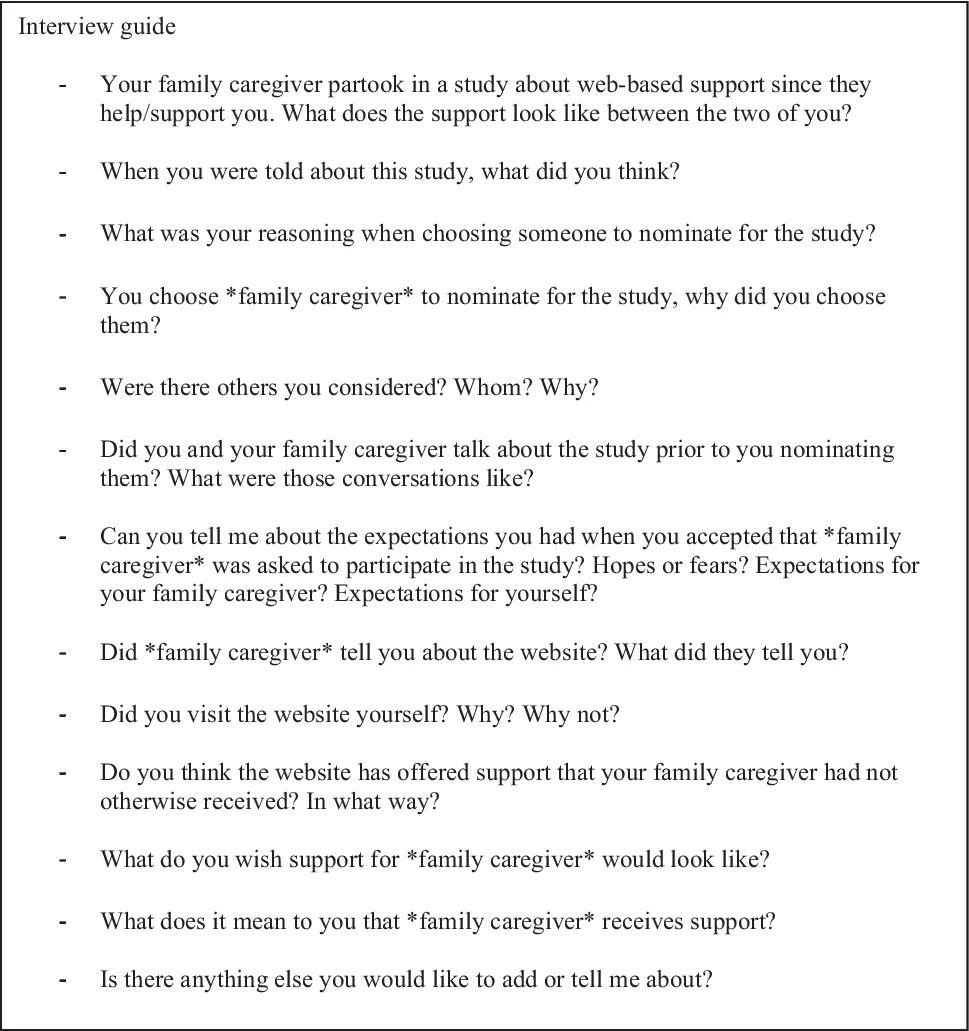
The interview guide

Individual, audio-recorded interviews were conducted (*n* = 22) using a semistructured interview guide (Fig. 2). As per the patients’ wishes, one interview was conducted face-to-face and 21 via telephone. The interview guide was used flexibly, allowing the interviewer to rephrase or switch the order of the questions based on the patient’s narrative. The interviewer was also sensitive towards each patient’s strength and stamina and how affected they were by their severe illness, thus adapting the interview’s length and tempo to their energy level. The sample size was continuously evaluated and the authors were guided by the principle of information power [18]. The core principle is that the more information power your data holds, the fewer participants you need, and hence, the interviews were regularly assessed for relevance and richness in order to determine when data generation should be terminated. The median interview was 40 min (range: 24–126 min). All interviews were conducted by the first author. The interviews were conducted between February and May of 2024.

### Data analysis

All interviews were transcribed verbatim and analyzed using interpretive description [16]. The interviews were listened to and read several times to attain a sense of data in its entirety. Segments from the interviews pertaining to the study aim were then extracted. This process was inclusively done to ensure that each segment’s surrounding context was not lost, which meant some segments were shorter (one or a few sentences) while others were longer (several sentences). Each segment then received a short description that captured the essence of what was being conveyed. In this process, the authors went back and forth between each segment and its placement in the interview to make sure its original context was not lost and the segment understood correctly. The short descriptions were then used to search for patterns within the content, whereafter tentative themes were formed. Again, the themes were looked at in relation to the interviews in total to ensure that the content of the interviews was conveyed with integrity. The analysis was mainly performed by the first (primary analyst) and last author (secondary analyst). However, remaining authors were regularly involved in the analysis by critical review of codes and tentative themes. All authors are registered nurses with clinical experience from palliative care as well as great experience of research in the field. There were a few occasions where the authors did not initially agree on codes or themes. These were then discussed in relation to the interviews until consensus was reached. Lastly, all themes were finalized in agreement between all authors.

### Ethical considerations

Written and oral informed consent was obtained from all participants. The study was performed in line with the principles of the Declaration of Helsinki. Approval was granted by the Swedish Ethical Review Authority (no.: 2022–02218-02 and 2022–06623-02).

## Results

The findings describe the patient group and the three themes that capture their expectations and motives when nominating a family caregiver for the study.

### Who were the participants?

The participants (*n* = 22) were a diverse group of patients who were eager to participate in the study and share their perspectives. Despite experiencing difficulties getting around physically, one patient insisted on travelling quite far to the university for the interview because partaking was important to him. Others returned the first author’s call from their hospital bed or ambulance ride, being admitted for, e.g., cancer treatment or blood transfusions but still wanting to do the interview. Further, some were adamant about completing the interview despite illness-related physical symptoms making it harder for them to breathe and talk. Characteristics-wise, the group consisted of equal proportions women and men (*n* = 11 + 11) from all of the five specialized palliative home care services in the study. Their median age was 73 years (range: 50–86). A majority had completed university-level studies (*n* = 14), were partners (*n* = 19) of the family caregiver who had participated in the intervention, and were diagnosed with cancer (*n* = 20) (Table 1).

**Table 1:** Overview of patient characteristics

Characteristic	
Age, median (range)	73 (50–86)
Sex	
Women, *n* (%)	11 (50.0)
Men, *n* (%)	11 (50.0)
Educational level	
9-year compulsory school, *n* (%)	1 (4.5)
High school-level studies, *n* (%)	7 (31.8)
University-level studies, *n* (%)	14 (63.6)
Diagnosis	
Cancer, *n* (%)	20 (90.9)
Neurological disease, *n* (%)	2 (9.1)
Relationship to family caregiver	
Partner, *n* (%)	19 (86.4)
Parent, *n* (%)	2 (9.1)
Friend, *n* (%)	1(4.5)

### Wishing for help—a feeling stemming from love, guilt and a sense of responsibility

The patients described different motives for nominating a family caregiver to participate in the study and common was a wish for them to receive support. They acknowledged that their illness affected not only themselves but also the persons important to them. Wishing for support for their family caregiver stemmed from love and concern, as the patients expressed occasionally feeling bad about the strain put on their family caregiver:

*“I felt bad that he didn’t get more support. And then you came along with your offer [the study] and I asked my husband how he felt about it, and he felt he could use some more support for himself. (…) Because… (…) in my worst dreams (…) I see myself on my very last breath in my hospital bed and that he’s supposed to take care of that and everything, and I just think… how is a simple human being supposed to handle all this that’s ahead of him? Then I think he needs support.”*

–Patient 2

Worry about their family caregiver put an emotional strain on them and they expressed a feeling that family caregivers’ needs and situation were sometimes overlooked by both healthcare providers and society in general. They felt responsible for supporting their family caregiver who in turn supported them and hoped the website would provide that. Offering support through nominating their family caregiver for the study was a way to express gratitude and give something back. For some patients, their family caregiver played a crucial role in, or was even a prerequisite for, them to be able to keep residing in their ordinary home as opposed to living in a care facility. They nominated their family caregiver to prevent the burden of their illness from becoming too heavy, which would in turn affect the patients themselves negatively by, e.g., them not being able to reside at home anymore:

*”It [support from my family caregiver] works very well in my case. But it’s very straining on her and it affects her part very much. (…) I’m very grateful and happy that she helps me. I’m completely… well the option would have been living in a nursing home or a residential facility, and that would have been incredibly sad.”*

–Patient 5

### Being mindful of which family caregiver to choose and not to coerce participation

Even though the patients were positive towards the study, they described being careful not to pressure their family caregiver into participating. They were mindful about the potential burden of the study despite perceiving it as potentially beneficial. When the family caregiver had been hesitant at first, the patients expressed how their own enthusiasm for the study eventually had inspired their family caregiver to participate:

*“I talked to her about it, and she agrees with me that… health care and that one can give back, and even if she hasn’t needed health care as much she’s seen me needing it. And she feels that… well, if I feel like this is good for her to do ‘then I’ll do it’.”*

–Patient 7

The most common relationship to the family caregiver was a partner, but there were also other relationships such as adult children or friends. The patients’ reasoning about nominating a family caregiver was tied to love, and choosing one’s partner was described as a given for those who did:

*“[I choose her] first of all because I love her. And second of all, I trust her 100 percent and she’s really good at everything. (…) Most of all because we’re so good together and I love her, we love each other.”*

–Patient 12

The patients nominated the family caregiver who provided most of the support for them and was the one most affected by their illness besides themselves. Those who chose their partner explained how the partner had naturally taken on the role as family caregiver and how they shared everyday lives:

*“Well it’s like this… how do I put this… the best life partner or life companion that I’ve had for so many years, there’s no one that knows me better than her. (…) She was my closest person, so that’s why [I choose her].”*

–Patient 21

Some patients described having considered different persons prior to nominating someone for the study, as they had to have certain qualities or skills. For example, they needed to be strong, resilient and grounded since the patients anticipated that they would face topics that might be difficult or unsettling in the study. Some had refrained from nominating certain close persons when they thought they were too busy or frail and they did not want to upset or burden them further:

*“You can put it like this, I have a good dialogue with my parents as well but they’re old and pa’ has had surgery and he’s broken and he’s starting to get senile, and ma’ is starting to get senile. I felt that, if you process them with questions and it gets wrong, (…) then she’ll walk around mulling it over and over in her head and think that… She makes a big deal out of small things, so I felt that’s probably not a good idea.*”

–Patient 15

### Expecting open conversations and help for family caregivers to face emotions

The patients expected the website to spark conversations between themselves and the family caregiver that they wanted but perceived as difficult and that not only the family caregiver but also they themselves had avoided in the past. They also hoped for the website to help the family caregivers face, and express, their emotions about life, death, and being a family caregiver. Further, they expressed that the website would be especially suitable when their family caregiver wanted to approach unsettling topics privately. The website would hopefully provide their family caregiver with much-needed support as a complement to regular healthcare:

*“It [the website] was about family caregivers of… (…) so I thought maybe he could get some help there, that’s what I thought. (…) If there was anything there that could be done that we didn’t know or don’t know so much about.”*

–Patient 16

When deciding to nominate their family caregiver, the patients expected the website to hopefully work as a way for them to also connect with others in similar situations. They hoped for the possibility for their family caregiver to learn about how others had solved or processed everyday problems. Furthermore, the patients expected the website to contain medical and practical information that their family caregiver would find relevant and helpful when supporting or caring for them. While not expecting it to replace human contact, they hoped it would be possible to tailor the content to each individual’s unique needs. One patient stressed the importance of individually tailored support and how the website might work especially well for, e.g., people without strong social networks:

*“I think it’s very individual. Some might think that it’s good that this [the website] exists because they have no one else to talk to or whatever, or think it’s difficult to talk to someone. Then maybe a website will work well. (…) If it was a lonely person then maybe… well then maybe you’ll sit down with a website. Or like I said, if you think it’s difficult to talk to friends and acquaintances (…) Then maybe it’s good that you can do it online. Everybody probably has the need to somehow talk about it or see what it’s like for others or get advice or anything.”*

–Patient 7

## Discussion

This study is unique in that it raises the patients’ voices in a randomized controlled trial evaluating web-based support for family caregivers, whereas previous research instead has focused the perspectives of, e.g., family caregivers and healthcare professionals on gatekeeping research from the patient in palliative care [19]. It provides novel insights into expectations and motives when nominating a family caregiver for participation in a web-based psychoeducational intervention among patients with predominantly advanced cancer. Recognizing the strain on the family caregiver by their illness and situation was an important reason for patients to nominate their family caregiver to partake. They were mindful when choosing and also refrained from nominating family caregivers whom they perceived as too fragile or busy. Further, they expected the intervention to spark conversations about life, death, and the current situation that they and their family caregiver had previously avoided.

The patients in the present study recognized how their illness affected their family caregiver and hence, wished for support for them via the intervention “narstaende.se.” They gave voice to the extensive strain put on family caregivers that has previously been shown in research [5–7]. They worried about their family caregiver’s wellbeing and also that they might eventually not be able to continue providing support for them at home. Remaining at home was evidently made possible by the support of family caregivers for some patients. In a society where a growing number of patients with life-threatening illness are being cared for at home [20–22], also at the end of life, it becomes increasingly important to identify the needs of family caregivers as it would benefit not only them but also in extension the patients.

Choosing a person to nominate for the study was done with utmost care by the patients. The most commonly chosen person was their partner, which might not be surprising given that research shows that partners predominantly act as the family caregiver [23]. There were also times when they refrained from nominating family caregivers they perceived as too frail or busy—a decision following a balancing act of wanting support for their family caregiver while also not wanting to add to their burden with an intervention. These insights into the reasoning of patients with life-threatening illness suggest that there probably are hard-to-reach family caregivers that could benefit from support when conducting intervention research targeting family caregivers or when offering support in clinical care. A climate where patients and family caregivers feel like they can, and want to, disclose and acknowledge their distress has shown to be important when fostering an environment where patients and family caregivers can support each other [24]. This type of transparency, encouraged by healthcare professionals already at clinical level, would likely shorten the path to the “hard-to-reach” family caregivers and put them on the radar for both clinical intervention (such as practical support or counselling by a social worker) and eligibility for research studies. Furthermore, the course of recruitment for research studies in palliative care would have to be evaluated. Perhaps recruiting family caregivers via the patient is not always the best course of action. Bulletins and invitations at bulletin boards at care units or via other channels (such as social media or official caregiver websites) may serve as a complement to reach less visible or obvious family caregivers.

In the present study, the patients expected the website to be a facilitator for conversations previously avoided. Talking about illness, end of life, and such matters has proven challenging for both patients [25, 26] with life-threatening illness and their family caregivers [26], as both wish to protect the other from the harm and discomfort such conversations might evoke. However, the present study shows that some patients actually do want to have these conversations with their family caregiver but doubt if or how to initiate them. Similarly, a previous study from the same research project where family caregivers and patients were interviewed in dyads following the family caregiver’s access to “narstaende.se” showed that the intervention helped family caregivers to have such conversations with the patient [27]. This shows that even though the web-based intervention “narstaende.se” was not accessed by the patients themselves, it still catalyzed conversations that both patient and family caregiver wanted but hesitated to start.

### Methodological considerations

Selection bias cannot be ruled out in this study, as in many studies involving patients at end of life. It is possible that the patients in worst condition decided against participating and we might therefore have missed important perspectives. The data generation was guided by the principle of information power [18]. The depth and richness of the interviews were regularly discussed among the authors, all with vast experience of qualitative research and analysis, and we were able to attain rich data covering various aspects of the study aim. Applying the principle, however, does not guarantee that new perspectives would not have come up had the data generation kept going. Researcher reflexivity [28] was maintained during the entire process of the study, from creating the interview guide to analysis of data and reporting of the results. We regularly discussed and brought awareness to, e.g., our own clinical experiences in the field and any preconceived ideas about the research topic. These ideas were scrutinized within the group and the critical discussions were kept alive all through the work with the study.

## Conclusion

Patients chose to nominate their family caregiver to the web-based intervention out of love, guilt, and responsibility. They expected open conversations with their family caregiver about their feelings, the patient’s illness, and the future. Furthermore, they hoped that the intervention would help family caregivers to face difficult emotions they might have previously avoided. The study highlights the care and consideration patients with life-threatening illness wish to show their family caregivers in a straining situation and also that they see potential benefits for themselves, such as the family caregiver being able to keep caring for them at home, when a family caregiver partakes in a support intervention. The study implies that support for their family caregivers is important for patients with life-threatening illness, also because it eases the strain on themselves. This reinforces the implications for support for family caregivers when organizing and providing care for patients with life-threatening illness.

## Data Availability

The data in the current study are not publicly available due to the General Data Protection Regulations and the Swedish Ethical Review Act, but are available from the corresponding author upon reasonable request.
